# Clinical and Serological Features of Patients Referred through a Rheumatology Triage System because of Positive Antinuclear Antibodies

**DOI:** 10.1371/journal.pone.0093812

**Published:** 2014-04-04

**Authors:** Christie Fitch-Rogalsky, Whitney Steber, Michael Mahler, Terri Lupton, Liam Martin, Susan G. Barr, Dianne P. Mosher, James Wick, Marvin J. Fritzler

**Affiliations:** 1 Faculty of Medicine, University of Calgary, Calgary, Alberta, Canada; 2 INOVA Diagnostics Inc., San Diego, California, United States of America; Keio University School of Medicine, Japan

## Abstract

**Background:**

The referral of patients with positive anti-nuclear antibody (ANA) tests has been criticized as an inappropriate use of medical resources. The utility of a positive ANA test in a central triage (CT) system was studied by determining the autoantibody profiles and clinical diagnoses of patients referred to rheumatologists through a CT system because of a positive ANA test.

**Methods:**

Patients that met three criteria were included: (1) referred to Rheumatology CT over a three year interval; (2) reason for referral was a “positive ANA”; (3) were evaluated by a certified rheumatologist. The CT clinical database was used to obtain demographic and clinical information and a serological database was used to retrieve specific ANA and/or extractable nuclear antigen (ENA) test results. Clinical information was extracted from the consulting rheumatologist's report.

**Results:**

15,357 patients were referred through the CT system; 643 (4.1%) of these because of a positive ANA and of these 263 (40.9%) were evaluated by a certified rheumatologist. In 63/263 (24%) of ANA positive patients, the specialist provided a diagnosis of an ANA associated rheumatic disease (AARD) while 69 (26.2%) had no evidence of any disease; 102 (38.8%) had other rheumatologic diagnoses and 29 (11%) had conditions that did not meet AARD classification criteria. Of ANA positive archived sera, 15.1% were anti-DFS70 positive and 91.2% of these did not have an AARD.

**Conclusions:**

This is the first study to evaluate the serological and clinical features of patients referred through a CT system because of a positive ANA. The spectrum of autoantibody specificities was wide with anti-Ro52/TRIM21 being the most common autoantibody detected. Approximately 15% of referrals had only antibodies to DFS70, the vast majority of which did not have clinical evidence for an AARD. These findings provide insight into the utility of autoantibody testing in a CT system.

## Introduction

The detection of anti-nuclear antibodies (ANA) has been established as an important adjunct to the diagnosis and classification of ANA-associated rheumatic diseases (AARD) such as systemic lupus erythematosus (SLE), systemic sclerosis (SSc), mixed connective tissue disease (MCTD), idiopathic inflammatory myopathies (IIM) and Sjögren's syndrome (SjS) [1]. Nevertheless, concerns have been raised about the ANA test as a screen for AARD [2], [3] and that positive tests inappropriately prompt unwarranted referrals from primary and secondary care physicians to tertiary care specialists [4]–[6]. Some concerns about ANA tests as an approach to screening for AARD are based on studies of the frequency of ANA in the healthy individuals [7] and calculations of pre-test probabilities and the clinical challenge of interpreting a positive test when the patient has no apparent evidence for a definitive diagnosis of, nor meets the classification criteria for, an AARD [3]–[5], [8].

The limitations of ANA and the related ENA tests have been offset by at least three key findings. First, for several decades it has been appreciated that some autoantibodies are highly specific for certain AARD [9], [10]. Hence, when disease specific autoantibodies, such as anti-dsDNA antibodies in SLE, anti-centromere antibodies in SSc and anti-Jo-1 antibodies in IIM are detected in the absence of diagnostic or classification criteria for these conditions the clinician is often uncertain about the advice to give to the referring physician or the patient. This issue is linked to a second key finding wherein it now well established that ANA and disease-specific autoantibodies can pre-date the clinical diagnosis of AARD by as many as two decades [11]–[13]. Thus, in the context of a person with a positive ANA where the specific autoantibody is known, the physician should take care before advising the patient that they do not have an AARD. Third, there is growing evidence that autoantibodies directed against the dense fine speckled 70 (DFS70) antigen without accompanying disease specific antibodies are rare in AARD and may be useful biomarker to rule out these conditions [14]–[17]. All three of these issues are of particular importance when patients are referred to a rheumatology central triage system because of a positive ANA test. Key questions are: 1) are such referrals inappropriate and a waste of health care resources, and 2) can the specificities of ANA and related autoantibodies inform the triage process to define the urgency of a specific referral to a specialist? Accordingly, the goals of this study were firstly to examine the ANA/ENA profiles of patients referred through a rheumatology central triage system; secondly, to determine if ANA/ENA of a given specificity were attended by a specific diagnosis and, thirdly, to determine the frequency of autoantibodies directed to DFS70 in a ANA referral cohort and elucidate the possible association of these antibodies to a specific diagnosis.

## Materials and Methods

### Ethics Statement

This study was approved by the University of Calgary Conjoint Health Research Ethics Board (Ethics ID#: E-24353). Under the terms of this approval, all patient records and information was anonymized and de-identified prior to analysis, precluding the requirement of written informed consent. All clinical investigation was conducted according to the principles expressed in the Declaration of Helsinki.

### Patients, Selection Criteria, Demographic and Clinical Information

We utilized an anonymous administrative database to evaluate the utility of autoantibody testing in the context of triage of referrals to the rheumatology service through a Central Referral and Triage (CReATe) service in the Calgary Health Region (Calgary, Alberta, Canada). This database includes the reason that the patient was referred for consultation, such as abnormal laboratory tests, signs or symptoms thought to suggest a rheumatic disease as well as baseline demographic data, working diagnoses and wait times. Of particular relevance to this study was the identification of patients referred to the rheumatology service because they had a positive ANA (ANA Referral Cohort: ARC). All individuals included in the ARC met the following criteria: (1) referred to Rheumatology Central Triage from in a sequential three year calendar time frame (n = 15,537); (2) reason for referral was a “positive ANA (n = 643); (3) were evaluated by certified rheumatology specialists at regional referral centres (n = 263) in Calgary, Alberta, Canada.

After the patient was seen by the consulting rheumatologist, a form was completed and information expressing the appropriateness of triage and final diagnosis was entered into the database. Final diagnoses were categorized as non-inflammatory disorders (e.g. fibromyalgia, osteoarthritis, tendonitis, and bursitis) or inflammatory diseases, including AARDs, rheumatoid arthritis (RA) and other systemic inflammatory arthritis. Accordingly, patients were grouped into three diagnostic categories (AARD, non-AARD and unresolved AARD). The AARD group included patients with a diagnosis of SLE, SSc, MCTD, IIM and SjS. The non-AARD included patients who had a definitive diagnosis but AARD was excluded. The third group (unresolved) consisted of patients without a final diagnosis or patients in which AARD insufficient classification criteria were not met and AARD could not be ruled out.

A second anonymous database that contained the serological data (see below) was extracted from the Mitogen Advanced Diagnostics Laboratory (MADL), Calgary, Alberta, Canada) master database. The two anonymous databases (CReATe and MADL) devoid of unique patient identifiers were merged using an anchored scrambled unique alphanumeric lifetime identifier (ULI) in order to maintain confidentiality.

A descriptive analysis of the combined dataset was performed, including the origin and number of referrals and autoantibody tests and results, triage categories, wait times and any delays in patient care resulting from requests for autoantibody tests. The number of patients referred because of a positive ANA test and their final diagnoses was tabulated. The primary analysis was the ability of an individual autoantibody to predict the diagnosis recorded by the consulting rheumatologist. This was gauged by determining the sensitivity, specificity, positive predictive value, negative predictive value and likelihood ratios for each test in relation to the final diagnosis, as well as the diagnostic categories described above. Secondary outcomes included the ability of individual or combined autoantibody results to predict the working diagnosis and triage category in order to assess how these results influence the triage process as it is currently designed. Agreement between the working diagnosis and the final diagnosis was assessed as a marker of referral quality.

### Autoantibody Testing

The MADL performs ANA and ENA tests for the Calgary Health Region and surrounding catchment areas. ANA testing (including pattern and titer assessment) utilized HEp-2 cell substrates (HEp-2000, ImmunoConcepts Inc., Sacramento, CA) to screen for autoantibodies by indirect immunofluorescence (IIF) at a dilution of 1/160 [7] on the sera stored at MADL. All available samples were also tested for ANA specificities included in the ENA screening panel (chromatin, ribosomal P, Sm, U1RNP (ribonucleoprotein), SS-A/Ro60, Ro52/TRIM21, SS-B/La, Scl-70 (topoisomerase I), Jo-1 (histidyl tRNA synthetase) by addressable laser bead immunoassay (FIDIS, TheraDiag: Paris, France), anti-centromere by IIF pattern on HEp-2 cells and dsDNA by the *Crithidia lucilliae* IIF test (ImmunoConcepts, Sacramento, CA) [18]. Last, antibodies to DFS70/LEDGF were detected by a chemiluminescent immunoassay (QUANTA Flash DFS70, INOVA Diagnostics, San Diego, CA)[16], [17].

### Statistics

The data were evaluated using the Analyse-it software (Version 1.62; Analyse-it Software, Ltd., Leeds, UK). Positive and negative likelihood ratios (LR) were calculated for the individual autoantibodies. For statistical analysis, patients were grouped into AARD (SLE, SSc, SjS, IIM and MCTD) and non-AARD patients. Individuals in which diagnosis was not fully established and verified or in which AARD could not be ruled out were excluded from respective analyses.

## Results

**Figure 1:**
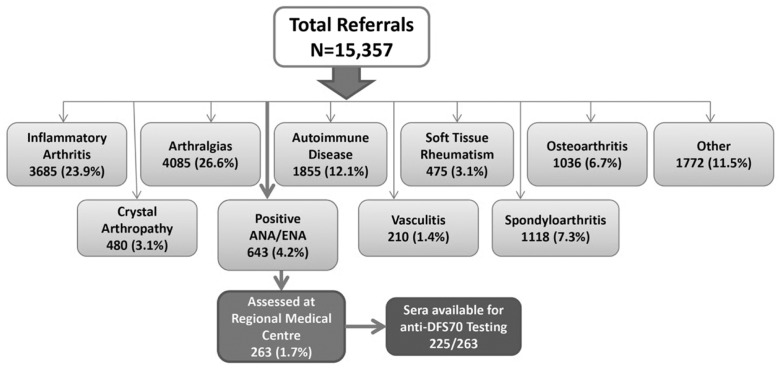
Derivation of the ARC via the diagnostic profile of 15,537 patients referred to rheumatology central triage over a three year audit period.

The clinical spectrum and referral diagnosis of all 15,357 patients referred to the Rheumatology service is represented in Figure 1. The majority of referrals were for evaluation of an inflammatory arthropathy (23.9%), arthralgia (26.6%) or a previously diagnosed AARD (12.1%), while smaller proportions were referred for consultation with respect to management of spondyloarthropathy (7.3%), osteoarthritis (6.7%), soft tissue rheumatism (3.1%) or vasculitis (1.4%). The ARC cohort of 263 patients from this referral group had an average age of 48.7 years (range 18–86, SD 83.53) and 92% were female. Approximately 68% of patients were referred from an urban center and 83.7% were referred by a family physician while the remainder were referred by subspecialist physicians: (in order of decreasing frequency) general internal medicine, neurology, respirology, otolaryngology, ophthalmology, dermatology, cardiology, hematology, obstetrics, general surgery, psychiatry. The mean interval from date of referral to when the patient was seen by the consultant was 165.4 days (range 25–551 days; SD 83.53).

Clinical information of the 263 ARC is summarized in Table 1. In total, 24% (n = 63) had a diagnosis of an AARD: SLE (9.1%; n = 24), SjS (9.1%; n = 24), SSc (2.3%; n = 6) and MCTD (1.9%; n = 5) followed by a spectrum of other AARDs or closely related conditions. Twenty-nine patients (11%) had equivocal evidence for an AARD and either did not meet sufficient criteria for classification or the consulting physician was unable to provide a definitive diagnosis (i.e. unresolved diagnosis): 13 (4.9%) undifferentiated connective tissue disease, 8 (3%) Raynaud's phenomenon, 5 (1.9%) inflammatory polyarthropathy and 3 (1.1%) discoid or cutaneous lupus. By comparison, 102 (38.8%) had a rheumatologic diagnosis but did not have an AARD, with osteoarthritis (9.9%), fibromyalgia (8.7%), arthralgia/myalgia (7.9%) and rheumatoid arthritis (RA) (2.3%) being the most common. Of note, 69 (26.2%) patients had no evidence for either an autoimmune or rheumatic disease. One hundred and sixteen patients (44.1%) of the ARC had a positive ENA test with the most common specificity being autoantibodies directed to Ro52/TRIM21 (n = 53; 45.7%), SS-A/Ro60 (n = 40; 34.5%), chromatin (n = 21; 18.5%), and SS-B/La (n = 19; 16.4%) (Table 2). The consultant's diagnosis linked to these anti-ENA autoantibodies is shown in Table S1 in File S1.

**Table 1:** Consultant's Opinion of 263 Patients in the ARC.

Consultant's Diagnosis	N	%
**AARD**	**63**	**24**
Systemic lupus erythematosus	24	9.1
Sjögren's syndrome	24	9.1
Systemic sclerosis	6	2.3
Mixed connective tissue disease	5	1.9
Drug-induced lupus	2	0.8
Dermatomyositis	2	0.8
**Unresolved AARD**	**29**	**11**
Undifferentiated connective tissue disease	13	4.9
Raynaud's phenomenon	8	3.0
Inflammatory polyarthropathy	5	1.9
Discoid and cutaneous lupus	3	1.1
**Other rheumatologic diagnoses**	**102**	**38.8**
Osteoarthritis	26	9.9
Fibromyalgia	23	8.7
Arthralgia/myalgia	21	7.9
Rheumatoid arthritis	6	2.3
Neuromuscular/neuropathy	6	2.3
Mechanical back pain	5	1.9
Vasculitis/polymyalgia rheumatica	2	0.8
Other*	13	4.9
**No findings for rheumatic or autoimmune disease**	**69**	**26.2**

*Other includes gout, tendonitis, bursitis, patellofemoral syndrome, palindromic rheumatism, spondyloarthropathy, psoriatic arthritis, unspecified polyarthropathy.

**Table 2:** Autoantibody Specificities of the 116 patients in the ARC with a positive anti-ENA*.

ENA Autoantibody**	N	%
Ro52/TRIM21	53	45.7
SS-A/Ro60	40	34.5
Chromatin	21	18.1
SS-B/La	19	16.4
Topoisomerase I (Scl-70)	17	14.7
U1 Ribonucleoprotein	17	14.7
Sm	14	12.1
Ribosomal P*	10	13.2
dsDNA*	10	13.2
Centromere	3	2.6
Jo-1	3	2.6
Unidentified	1	0.8

Note: 116/263 patients referred with a positive ANA had a detectable ENA autoantibody. Some patients had more than 1 autoantibody, hence % totals will not  = 100.

*results available for 76 samples.

**Clinical diagnoses associated with specific autoantibodies see Table S1 in File S1.

The IIF results of the ARC showed that the three most common primary ANA IIF patterns were speckled (n = 158; 60.1%), nucleolar (n = 66; 25.1%), and homogeneous speckled (n = 55; 20.9%), with titers ranging from 1/160-1/5120 (Table S2 in File S1). Other IIF staining patterns included cytoplasmic (27.4%), multiple nuclear dots (26%), nuclear matrix and centromere (7.6%) and other less common patterns (Table S2 in File S1).

### Anti-DFS70 antibodies

**Figure 2:**
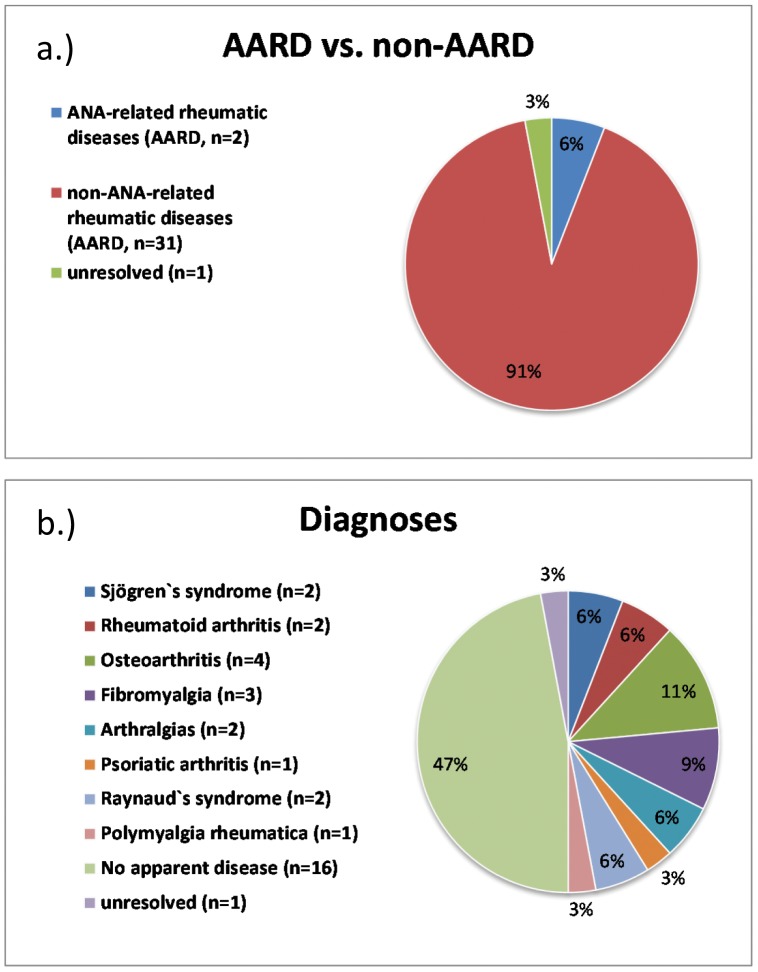
Clinical diagnoses of 34 patients with anti-DFS70 antibodies. Panel a.) frequency (%) of ANA-associated rheumatic diseases (AARD) and non-AARD. One patient with undifferentiated connective tissue disease did not match AARD or non-AARD and was classified as “unresolved”. Panel b.) frequency of diagnoses of the patients with anti-DFS70 antibodies is shown.

**Figure 3:**
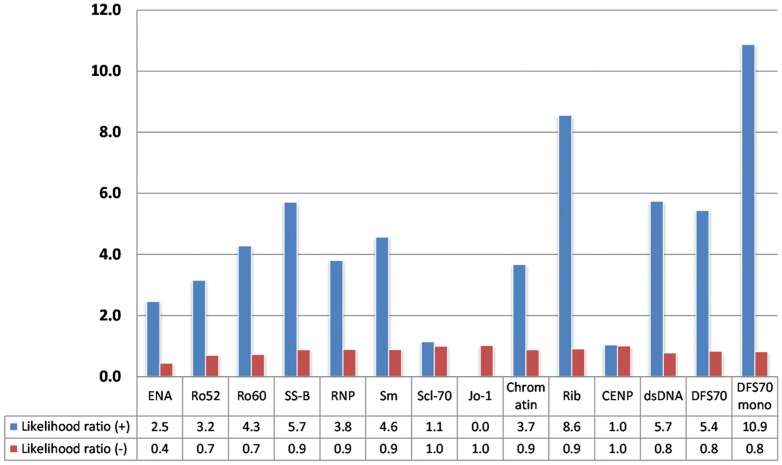
Likelihood ratios differentiate AARD from non-AARD in ANA positive patients (n = 208, 55 excluded due to missing results; dsDNA based on 76 samples). NOTE: Anti-DFS70 positivity was indicative for non-AARD. ‘DFS70 mono’ represents patients that have anti-DFS70, but no other detectable autoantibody.

Thirty-four of the 225 (15.1%) archived sera tested positive for anti-DFS70 antibodies (Figure 2) and was the sole autoantibody detected in 24/34 (70.6%) of the sera. Among the anti-DFS70 antibody positive cohort, 33 had a definite diagnosis (one was unresolved) and of those 31/33 (93.9%) had no evidence of or fulfilled classification criteria for an AARD (Figure 3). The two patients with an AARD had SjS (one with coexistent anti-Ro52/TRIM21 antibodies). Sixteen (47.1%) anti-DFS70 positive patients had no evidence for any disease although three of these had autoantibodies typically associated with an AARD: one with anti-Scl-70/topo I and two with anti-chromatin antibodies. Other diagnoses in the anti-DFS70 antibody positive patients included Raynaud's phenomenon, osteoarthritis, RA, fibromyalgia and individual patients with polymyalgia rheumatica and psoriatic arthritis (Figure 2).

**Figure 4:**
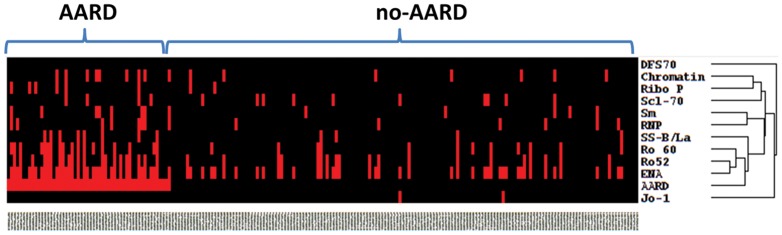
Supervised cluster analysis with patients sorted according to the presence or absence of ANA associated rheumatic disease (AARD) was performed with antibodies to extractable nuclear antigens (ENA), chromatin, ribosomal P and to DFS70. ENA, Ro52/TRIM21, SS-A/Ro60 and SS-B/La cluster most closely with AARD whereas DFS70 antibodies and Jo-1 demonstrate high distance clustering.

To analyze if autoantibodies might provide value in determining the urgency of referral we calculated the LRs for all anti-ENAs and for anti-DFS70 antibodies (Figure 3). The LR+ for AARD showed significant variations ranging from 0.0 (anti-Jo-1 antibodies) to 8.6 (anti-Ribo-P antibodies). The LR+ for non-AARD for anti-DFS70 antibodies was 5.4 and when considered in conjunction with other autoantibodies (no coexisting antibody/monospecific for anti-DFS70 antibodies) the LR+ increase to 10.9. Clarity on the usefulness of ANA, anti-ENA and anti-DFS70 antibody testing was illustrated by a supervised cluster analysis (Figure 4).

## Discussion

### Diagnosis of Patients with Positive ANA/anti-ENA antibodies

One of the time honored adjuncts to diagnosis of AARD or the broader spectrum of systemic autoimmune rheumatic diseases is autoantibody testing. In most of these conditions, autoantibodies are included as part of validated disease classification criteria [19]–[22]. Accordingly, on the backdrop of a wide range of protean clinical signs and symptoms, autoantibody tests, and the ANA test in particular, are often used as a screening tool for AARD by referring physicians [3], [5], [23]–[25]. The referral of patients with positive ANA and/or ENA to tertiary care specialists is a common practice that has met with some criticism [3], [5], [23], [26], [27], with one claim being that because pre-test probabilities of AARD is low, such referrals are unnecessary and a burden on the health care system resulting increased wait times for rheumatology consultations. This can be complicated when the referring physician has told the patient that they do or do not have an AARD. Such misdiagnoses (over-diagnosis or under-diagnosis) can have emotional and insurability consequences for the patient and a serious negative impact on health care resources, including inappropriate or delays in appropriate therapy culminating in poor clinical outcomes, such as irreversible organ damage or failure [5], [28]–[30]. These issues also have ethical implications [31] and algorithms for how cost-effective and rational autoantibody testing could be harmonized and developed into a standardized schema is gaining international attention [23], [24], [32], [32]–[37].

In 1989 Shiel and Jason [38] reported that patients referred to a community rheumatologist because of a positive ANA with a titer of 1/40 or greater (comprising 8.8% of all referrals compared to 4.1% in our study) received a specific diagnosis in 86.6% and of those, 51.4% had a connective tissue disease [38]. An interesting study by Narain et al [5] reported that of 263 patients referred with a presumptive diagnosis of SLE, 48% received a diagnosis of other conditions and 29% were seropositive for ANA but did not have autoimmune disease. Disconcertedly, 39 patients who were seropositive for ANA but had no autoimmune disease had been treated with corticosteroids at dosages as high as 60 mg/d. By comparison, a more recent study by Abeles and Ables [27] found that none of the patients referred to a specialist clinic with an ANA titer of <1/160 had an AARD, and like previous studies [39], the majority had soft tissue rheumatism and other pain syndromes. In our study, where the screening ANA test was performed at a serum dilution of 1/160, approximately 27% of patients referred because of a positive ANA/ant-ENA antibody test had an AARD, approximately 10% had equivocal evidence for AARD, and the remainder had a wide variety of rheumatic (i.e. rheumatoid arthritis) and non-rheumatic conditions. In another study of 50 ANA positive patients that did not fulfill sufficient criteria for classification of SLE, anti-Ro60/SS-A and anti- La/SS-B antibodies were the most common detectable anti-ENA antibodies (6%) using a limited diagnostic profile and while none of the patients with leukopenia, thrombocytopenia, fatigue and arthralgia did not evolve to a full diagnosis of SLE, the authors concluded that ANA positivity connoted a form of systemic autoimmunity [40]. By comparison, patients with arthritis, rash, Raynaud's phenomenon and anti- Ro60/SS-A autoantibodies did evolve to SLE. These observations are interesting in the context of our study since we have initiated a longitudinal study to determine which ACR patients with a positive ANA or anti-ENA antibody test that did not meet criteria for an AARD evolve into an AARD such as SLE.

The serum dilution at which screening ANA tests are performed lacks uniformity from laboratory to laboratory. A study by an international advisory committee comprised of representatives from fifteen participating laboratories concluded that the ANA screening dilution should be set at 1/160 to increase the specificity of the ANA test [7]. Nevertheless, as exemplified by the report of Abeles and Abeles [27] and others [38], many laboratories continue to test sera at lower screening dilutions. Based on our study it appears that even if the screening serum dilution is set at 1/160, more than one-half of patients do not have sufficient clinical evidence or classification criteria to make a diagnosis of an AARD at the time of evaluation by the consulting rheumatologist.

There is hope that a more thorough understanding of ANA and anti-ENA antibody specificities can help address some of the inherent shortcomings of ANA and anti-ENA antibody specificity and sensitivity. For example, mounting evidence indicates that the presence of anti-DFS70 antibodies is a useful biomarker found in ANA positive individuals the vast majority of whom did not have an AARD [14]–[17]. In the present study, approximately 15% of the ARC had antibodies directed to DFS70 and in the majority (73.5%) it was the only detectable autoantibody. Of these anti-DFS70 positive patients, only 2/33 (6.1%) had an AARD while approximately one-third had either no evidence for a disease or a variety of other non-AARD diagnoses such as osteoarthritis and fibromyalgia. In summation, our findings support previous studies indicating that anti-DFS70 antibodies tend to exclude the diagnosis of AARD [16], [17], [41] and the observation that the majority are “monospecific” for anti-DFS70 antibodies add further evidence to the potential value of routinely testing for these autoantibodies.

### Use of Autoantibody Profiles to inform Patient Triage and Referral Systems

This is the first published study to evaluate the clinical diagnoses and serological parameters of patients referred to rheumatologists through a central triage system because of a positive ANA test. Access to rheumatologists is often inadequate to meet patient needs due to population growth, aging of the population, and a declining supply of specialists [42]. In our study, the average waiting time from referral to when the patient was seen by the consulting rheumatologist was 165 days (> five months). Although it is intuitive, there is evidence that an early and accurate diagnosis of patients with rheumatic diseases improves health outcomes while reducing health care costs [35], [43]–[48]. In our study, the vast majority (∼80%) of referrals for a positive ANA was from general practitioners (GPs) and at least one study has shown that the threshold used by GPs for referral to rheumatologists is low leading to lengthy wait times [49]. It was suggested that more frequent use of telephone consultations and improved diagnostic skills of GPs may alleviate the wait times. Another suggested approach was to use registered nurses and GPs themselves to do the triage [50], an approach that had a degree of diagnostic accuracy rivaling that of experienced rheumatologists. The limitation to this approach is that it seems an assumption that GPs are available for such activity because they are not as busy as rheumatologists. Using appropriately trained registered nurses and/or other allied health care personnel is the approach that we have taken to screen and adjudicate referrals as a “team” approach as recommended by others [51].

A key component to improving timely, efficient and appropriate access to specialist consultation and care is the ability of the triage team to assign a level of urgency to the referral. An effective and efficient triage system requires the formulation of a working diagnosis based on the information included in the referral letter [52]. This may be particularly difficult in rheumatology, as there are few diagnostic tests that can firmly establish a diagnosis. The diagnosis of AARD relies on a complete history, physical examination and investigations [34], and depends on the expertise of the care provider. In the context of rheumatology referrals because of a positive ANA, our data suggest that patients with isolated anti-DFS70 autoantibodies do not require urgent referral to a specialist, whereas patients with anti-ENA antibodies and other AARD-specific autoantibodies, where early and accurate diagnosis is a goal, require rapid assessment for an AARD.

### Limitations

This study was a cross-sectional study without patient follow-up to determine the longer term outcome of patients. The utility of ANA testing in a triage or referral setting or in diagnostic algorithms is complicated by a number of factors, most notably that autoantibodies often precede symptoms and overt evidence of disease by as much as two decades [53], [54]. While this phenomenon is well established in medical literature, many physicians have yet to embrace the concept in their clinical evaluation of patients. To help understand the use of autoantibodies as predictors of diseases, the ARC in this study is being followed for long term clinical outcomes.

In addition, this study did not assess the economic impacts of the referral system. The cost effectiveness of autoantibody testing and its effective application to the clinical setting is clearly required [35], and if there were more effective ways to triage patients that will never develop an AARD, cost savings could be significant. To date, rather than a holistic cost-benefit analysis, cost analyses have tended to focus on the cost of the ANA test itself and related algorithms [3], [23], [36], [37]. Of note, it has already been suggested that the detection of anti-DFS70 autoantibodies may result in meaningful cost savings [14], [55] by the potential of reducing the additional testing in search of establishing the diagnosis of a systemic autoimmune rheumatic disease and in some cases the initiation of inappropriate therapy [5].

### Future Considerations and Conclusions

With the emergence of novel biomarkers for AARD, it is thought that disease prevention and morbidity amelioration by establishing an early and accurate diagnosis will likely not rely on a single or any one class of biomarkers (i.e. autoantibody, genetic, metabolomic). Rather than a single autoantibody marker (i.e. anti-Scl-70/topo I, anti-dsDNA, anti-DFS70) it is likely that multiplexed autoantibody profiles will provide more important clinical information in the future. Our study suggests that the fine specificities of ANA and other autoantibodies are important to decide whether or not an urgent referral is needed. For example, based on other corroborating studies [15]–[17], our present study indicates that if the patient has isolated anti-DFS70 antibodies, then urgent referral is likely not required. However, if patients have certain anti-ENA, such as anti-dsDNA or anti-ribosomal P, then more urgent assessment is indicated.

## Supporting Information

File S1 **File contains Table S1 and Table S2.** Table S1: Consultant Diagnosis of anti-ENA Antibody Positive Individuals. Table S2: Indirect Immunofluorescence Patterns of Sera from Patients in the Referral Cohort.
(DOCX)
