# Supplementary material for: Clinical and Serological Features of Patients Referred through a Rheumatology Triage System because of Positive Antinuclear Antibodies
Source: PLoS One. 2014 Apr 4;9(4):e93812. doi: 10.1371/journal.pone.0093812 (PMC3976309; doi:10.1371/journal.pone.0093812)
Supplement: File S1 — File contains Table S1 and Table S2. Table S1: Consultant Diagnosis of anti-ENA Antibody Positive Individuals. Table S2: Indirect Immunofluorescence Patterns of Sera from Patients in the Referral Cohort. (DOCX) [file pone.0093812.s001.docx]

**Table S1: Consultant Diagnosis of anti-ENA Antibody Positive Individuals**

| **Autoantibody/Titer (n)*** | **Consultant’s Diagnosis** |
| --- | --- |
| **Ro52/TRIM21 (53)** | |
| Low (10) | 2-arthralgias, 1- SLE, 3-no autoimmune disease, 1-RA, 1-palindromic syndrome, 1-SjS, 1-UCTD |
| Med (13) | 8-SjS, 2-no autoimmune disease, 1-cutaneous lupus, 1-SLE, 1-UCTD |
| High (30) | 2-inflammatory polyneuropathy, 4-SLE, 10-SjS, 2-OA, 1-FM, 1-MCTD, 1-UCTD, 3-arthralgias, 3-no autoimmune disease, 1-gout, 1-OA, 1-inflammatory arthritis |
| **SSA/Ro 60 (40)** | |
| Low (6) | 2-FM, 2-SLE, 1-RA, 1-no autoimmune disease |
| Med (9) | 1-SjS, 2-OA, 1-MCTD, 1-arthralgias, 1-SLE, 1-UCTD, 2-no autoimmune disease |
| High (25) | 1- inflammatory polyneuropathy, 13- SjS, 3-SLE, 2-cutaneous lupus, 1-gout, 1-OA, 1-polyarticular inflammatory arthritis, 3-no autoimmune disease |
| **Chromatin (21)** | |
| Low (8) | 2-arthralgias, 1-neuromuscular disease, 1-UCTD, 2-no autoimmune disease, 2-SLE |
| Medium (8) | 1- no autoimmune disease, 3-SLE, 3-SjS, 1-arthralgias |
| High (5) | 1-polyarthritis, 1-SLE, 1-SSc, 1-no autoimmune disease, 1-FM |
| **SSB/La (19)** | |
| Low (5) | 1-FM, 2-OA, 1-cutaneous lupus, 1-UCTD |
| Med (3) | 1-SLE, 1-arthralgias, 1- UCTD |
| High (11) | 4- SjS, 1-arthralgias, 1-UCTD, 1-OA, 1-inflammatory arthritis, 2-SLE, 1-no autoimmune disease |
| **Topo I (17)** | |
| Low (12) | 4-no autoimmune disease, 1-SSc, 2-FM, 1-UCTD, 2-OA, 1-SLE, 1- Raynaud's phenomenon |
| Med (1) | 1-inflammatory arthritis |
| High (4) | 1-SSc, 1-OA, 1-SLE, 1-no autoimmune disease |
| **RNP (17)** | |
| Low (5) | 2-no autoimmune, 1-discoid lupus, 1-SjS, 1-SLE |
| Med (4) | 1-MCTD, 1-SjS, 1-SLE, 1-RA |
| High (8) | 1-MCTD, 4-SLE, 1-no autoimmune disease, 1-palindromic rheumatism, 1 UCTD |
| **Sm (14)** | |
| Low (9) | 6-SLE, 1-UCTD, 1-SjS, 1-no autoimmune disease |
| Med (4) | 1-MCTD, 2-no autoimmune disease, 1-discoid lupus |
| High (1) | 1-SLE |
| **Ribosomal P (10)** | |
| Low (6) | 1-no autoimmune disease, 3-SLE, 1-SSc, 1-arthralgias |
| Med (2) | 1-SLE, 1-no autoimmune disease |
| High (2) | 2-SLE |
| **dsDNA (10)** | |
| Positive by Crithidia lucilliae IIF assay | 2-SLE, 1-SSc, 2-SjS, 2-no autoimmune disease, 1-arthralgias, 1-DIL, 1-UCTD  Note: 76 sera tested |
| **Jo-1 (3)** | |
| Low (3) | 1-FM, 2-no autoimmune |

Abbreviations: DIL, drug induced lupus; dsDNA, double stranded DNA (Crithidia assay); FM, fibromyalgia; IIF, indirect immunofluorescence; Jo-1, histidyl tRNA synthetase; MCTD, mixed connective tissue disease; OA, osteoarthritis; RA, rheumatoid arthritis; RNP, ribonucleoprotein; SjS: Sjögren’s syndrome; SLE, systemic lupus erythematosus; Sm, Smith antibody; SSA, Sjögren’s syndrome antigen A (Ro60); SSB, Sjögren’s syndrome antigen B (La); SSc, systemic sclerosis; Topo I, topoisomerase I (Scl-70); TRIM, tripartite motif; UCTD, undifferentiated connective tissue disease.

* Low, Medium, High = semi-quantitative levels (titers) of autoantibodies

**Table S2: Indirect Immunofluorescence Patterns of Sera From Patients in the Referral Cohort**

| **IIF Pattern (n)** | **% *** | **Titer Range** |
| --- | --- | --- |
| Speckled (158) | 60.1 | 1/160 – 1/5120 |
| Cytoplasmic (72) | 27.4 | 1/160 – 1/2560 |
| Nucleolar (66) | 25.1 | 1/160 – 1/5120 |
| Homogenous & speckled (55) | 20.9 | 1/160 – 1/5120 |
| Multiple nuclear dots (26) | 9.9 | 1/320 – 1/5120 |
| Nuclear matrix (20) | 7.6 | 1/160 – 1/5120 |
| Centromere (19) | 7.2 | 1/320 – 1/5120 |
| Nuclear envelope (18) | 6.8 | 1/160 – 1/1280 |
| Homogeneous (17) | 6.5 | 1/160 – 1/5120 |
| Mitotic spindle apparatus (13) | 4.9 | 1/160 – 1/1280 |
| Mitochondria (3) | 1.1 | 1/320 – 1/1280 |

* Total does not equal 100% because some sera had more than one IIF pattern.
